# Reactive oxidant species induced by antifungal drugs: identity, origins, functions, and connection to stress-induced cell death

**DOI:** 10.3389/fcimb.2023.1276406

**Published:** 2023-10-12

**Authors:** Irene Gonzalez-Jimenez, David S. Perlin, Erika Shor

**Affiliations:** ^1^ Center for Discovery and Innovation, Hackensack Meridian Health, Nutley, NJ, United States; ^2^ Medical Sciences, Hackensack Meridian School of Medicine, Nutley, NJ, United States; ^3^ Lombardi Comprehensive Cancer Center and Department of Microbiology and Immunology, Georgetown University, Washington, DC, United States

**Keywords:** reactive oxidant species, ROS, antifungal drugs, fungal pathogens, programmed cell death, azoles, echinocandins, polyenes

## Abstract

Reactive oxidant species (ROS) are unstable, highly reactive molecules that are produced by cells either as byproducts of metabolism or synthesized by specialized enzymes. ROS can be detrimental, e.g., by damaging cellular macromolecules, or beneficial, e.g., by participating in signaling. An increasing body of evidence shows that various fungal species, including both yeasts and molds, increase ROS production upon exposure to the antifungal drugs currently used in the clinic: azoles, polyenes, and echinocandins. However, the implications of these findings are still largely unclear due to gaps in knowledge regarding the chemical nature, molecular origins, and functional consequences of these ROS. Because the detection of ROS in fungal cells has largely relied on fluorescent probes that lack specificity, the chemical nature of the ROS is not known, and it may vary depending on the specific fungus-drug combination. In several instances, the origin of antifungal drug-induced ROS has been identified as the mitochondria, but further experiments are necessary to strengthen this conclusion and to investigate other potential cellular ROS sources, such as the ER, peroxisomes, and ROS-producing enzymes. With respect to the function of the ROS, several studies have shown that they contribute to the drugs’ fungicidal activities and may be part of drug-induced programmed cell death (PCD). However, whether these “pro-death” ROS are a primary consequence of the antifungal mechanism of action or a secondary consequence of drug-induced PCD remains unclear. Finally, several recent studies have raised the possibility that ROS induction can serve an adaptive role, promoting antifungal drug tolerance and the evolution of drug resistance. Filling these gaps in knowledge will reveal a new aspect of fungal biology and may identify new ways to potentiate antifungal drug activity or prevent the evolution of antifungal drug resistance.

## Introduction: reactive oxidant species and fungal pathogens

Reactive oxidant species (ROS) are a host of unstable molecules that are highly reactive with other molecules in their vicinity, sometimes with detrimental consequences (Schieber and Chandel, 2014; Sies et al., 2017; Sies et al., 2022). Most ROS are oxygen- or nitrogen-based, but can also contain chlorine, sulfur, etc., and can be either free radicals (e.g., hydroxyl radical ^•^OH, superoxide O_2_^•-^, or nitric oxide NO^-•^) or non-free radicals (e.g., hydrogen peroxide H_2_O_2_ or hypochlorite HOCl). ROS are produced by all living organisms, either as byproducts of cellular metabolism or synthesized by specialized enzymes, such as NADPH oxidases and nitric oxide synthases. For several decades after their discovery in the 1960s, ROS were considered to be exclusively detrimental to cells because of the oxidative damage they could cause to cellular macromolecules, including DNA, proteins, and lipids. However, eventually it became apparent that the consequences of ROS could be either detrimental or beneficial, depending on the specific molecules produced and their levels (Winterbourn, 2008; Finkel, 2011; Schieber and Chandel, 2014; Sies et al., 2017; Lennicke and Cochemé, 2021; Sies et al., 2022). Therefore, the current prevailing paradigm is that ROS are integral components of normal cellular signaling and functions, and the formerly widely used concept of “oxidative stress” has been refined to include “oxidative distress”, wherein ROS are present at high and damaging levels, and “oxidative eustress”, wherein ROS are present at levels supporting normal cellular homeostasis (Sies et al., 2017).

In some contexts, however, the damaging capacity of ROS is integral to their biological function. The best studied example of this is the capacity of immune cells to produce high levels of ROS via specialized enzymes to kill pathogenic microbes. Conversely, microbial pathogens, including fungi, have evolved a range of defense mechanisms that allow them to counteract and survive the ROS onslaught imposed by host immunity (Warris and Ballou, 2019; Yaakoub et al., 2022). Furthermore, fungal cells themselves have been shown to produce endogenous ROS that play important roles in promoting fungal morphogenesis, including at the host-pathogen interface (Rossi et al., 2017). It is in the context of host-pathogen interaction that the connection between fungal pathogens, ROS, and fungal antioxidant systems has been largely studied and discussed (Warris and Ballou, 2019; Yaakoub et al., 2022). Interestingly, over the last decade, a number of studies have shown that ROS are also produced by fungal cells in response to antifungal drug treatment (**Table 1**). However, despite the accumulating evidence for antifungal drug-induced ROS formation, the implications of this phenomenon are still largely unknown. In this review we summarize the evidence for antifungal drug-induced ROS formation in fungal cells, outline what is known about their chemical nature, molecular origin, and possible functions, highlight the large gaps in knowledge in these three areas, and outline the approaches that could fill those gaps. The focus of this review is on the most prevalent fungal pathogens of humans that cause invasive infections associated with high mortality (namely, *Candida, Aspergillus*, and *Cryptococcus* species), and we will discuss evidence for ROS production by fungi specifically in response to antifungal drugs currently used in the clinic to treat invasive fungal infections: azoles, polyenes, and echinocandins. Finally, we will discuss these observations in the broader context of ROS formation as part of the fungal programmed cell death (PCD) cascade triggered by various types of environmental stress.

**Table 1:** Reports of ROS formation in fungal pathogens in response to antifungal drugs.

		*Candida*					*Nakaseomyces*	*Cryptococcus*		*Aspergillus*
		*C. albicans*		*C. parapsilosis*	*C. tropicalis*	*C. krusel*	*N. glabratus*	*C. neoformans*	*C. gatii*	*A. fumigatus*
Azoles	Fluconazole	Belenky et al. (2013); Muñoz-Megías et al. (2023)	Kobayashi et al. (2002)				Mahl et al. (2015)	Peng et al. (2018); Dbouk et al. (2019)	Ferreira et al. (2013)	
	Itraconazole	Peng et al. (2018)		Muñoz-Megías et al. (2023)					Ferreira et al. (2013)	Shekhova et al. (2017)
	Miconazole	Kobayashi et al. (2002); Belenky et al. (2013); Muñoz-Megías et al. (2023)								
Polyenes	Amphotericin B	Phillips et al. (2003); Belenky et al. (2013); Mesa-Arango et al. (2014); Guirao-Abad et al. (2017)		Mesa-Arango et al. (2014)	Mesa-Arango et al. (2014)	Mesa-Arango et al. (2014)	Mesa-Arango et al. (2014)	Sangalli-Leite et al. (2011); Mesa-Arango et al. (2014)	Ferreira et al. (2013); Mesa-Arango et al. (2014)	Shekhova et al. (2017)
Echinocandins	Caspofungin	Kobayashi et al. (2002); Hao et al. (2013)					Garcia-Rubio et al. (2021)			Shekhova et al. (2008); Satish et al. (2019)
	Micafungin	Guirao-Abad et al. (2017)					Garcia-Rubio et al. (2021)			Satish et al. (2019)

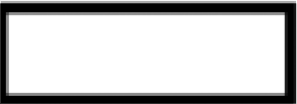
 ROS not tested.

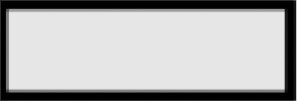
 No ROS increase detected.

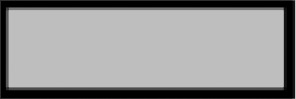
 ROS increase detected.

## ROS form in fungi during antifungal drug exposure

Three major classes of antifungal drugs are currently used clinically to treat invasive fungal infections – azoles, echinocandins, and polyenes – and increased ROS production has been detected in fungal cells in response to all of these drug classes (**Table 1**). In this section we briefly describe the antifungal mode of action for each of these drug classes and summarize the available experimental evidence for ROS induction in response to treatment. In the following sections we will discuss the identity, cellular origins, and functions of these ROS, focusing in particular on the large gaps in knowledge in each of these areas and possible approaches to fill those gaps.

Azoles are a frontline antifungal drug class that works by inhibiting the biosynthesis of ergosterol, an essential component of fungal cellular membranes. Most azoles exert static effects in yeasts (*Candida*, *Nakaseomyces*, *Cryptococcus*) but are cidal in molds, such as *Aspergillus*. Evidence of ROS formation in response to azole exposure has been reported in both yeasts and molds (**Table 1**). Interestingly, in *Candida*, the most robust and reproducible evidence for ROS formation has been obtained in response to fungicidal azoles: miconazole and itraconazole (Kobayashi et al., 2002; Francois et al., 2006; Belenky et al., 2013; Lee and Lee, 2018; Muñoz-Megías et al., 2023). In contrast, investigating whether *C. albicans* induces ROS in response to fluconazole, which is fungistatic in *Candida*, has produced conflicting results (Kobayashi et al., 2002; Francois et al., 2006; Belenky et al., 2013) (**Table 1**). Indeed, some studies have explicitly noted that ROS were robustly induced in response to fungicidal drugs but not fungistatic ones (Francois et al., 2006; Belenky et al., 2013). On the other hand, ROS increases have been detected in response to fluconazole in *Nakaseomyces glabratus* (formerly and still frequently referred to as *Candida glabrata*) (Mahl et al., 2015) and *Cryptococcus neoformans* (Peng et al., 2018; Dbouk et al., 2019) (but not *C. gattii* (Ferreira et al., 2013)), i.e., species in which fluconazole is fungistatic. Thus, the relationship between cidality and ROS induction is suggested by some data but does not appear to be universal. The mechanisms proposed to link azole action to ROS formation are not fully understood but have invoked mitochondrial membrane perturbation by the azoles, resulting in respiratory chain defects, electron leakage, and increased ROS production (Shekhova et al., 2017). Also, as discussed at length below, azole-mediated disruption of mitochondrial integrity may trigger the fungal PCD response, in which ROS are a key intermediate (Ramsdale, 2008; Strich, 2015).

Polyenes are the oldest antifungal agents in clinical use, which exert their antifungal action by binding to ergosterol in the fungal membranes, resulting in loss of membrane integrity. These drugs are not used as frontline agents due to their high incidence of toxic side effects. Nevertheless, polyenes, of which amphotericin B is the most frequently used member, are an important part of our antifungal drug armamentarium due to high intrinsic or increasing acquired resistance to frontline antifungals (azoles and echinocandins) in some fungal species (Perlin et al., 2015; Gow et al., 2022). Amphotericin B is fungicidal against all fungal species, at least *in vitro*, and it has been shown to induce robust ROS formation in all yeast and mold species examined to date, including several *Candida* species, *N. glabratus*, *C. neoformans* and *C. gattii*, and *Aspergillus fumigatus* (Phillips et al., 2003; Sangalli-Leite et al., 2011; Belenky et al., 2013; Ferreira et al., 2013; Mesa-Arango et al., 2014; Guirao-Abad et al., 2017; Shekhova et al., 2017) (**Table 1**). How amphotericin B induces ROS production is not clear, but mitochondrial membrane disruption has been proposed as the initiating event (Shekhova et al., 2017), and several studies have proposed that this antifungal agent has multiple mechanisms of action, whereby its capacity to induce oxidative distress is separate from its capacity to disrupt fungal membranes (Sangalli-Leite et al., 2011; Ferreira et al., 2013). There is also significant evidence that fungal cells exposed to amphotericin B exhibit multiple features of PCD, including ROS formation, nuclear fragmentation, and externalization of phosphatidylserine on plasma membrane (Phillips et al., 2003; Mousavi and Robson, 2004), but whether this PCD is triggered by mitochondrial membrane disruption or other drug effects is not yet understood.

Echinocandins are a frontline antifungal drug class that acts by inhibiting 1,3-β-glucan synthase, the enzyme that synthesizes an essential component of the fungal cell wall, and thus disrupting fungal cell wall integrity. The first echinocandin introduced into the clinic (in the early 2000s) was caspofungin, but other members of the class, first micafungin and then anidulafungin, have been approved for clinical use since then. Echinocandins are cidal in *Candida*, static in molds, such as *Aspergillus*, and inactive against *Cryptococcus*. Of the three drug classes discussed in this review, the effect of echinocandins on fungal ROS formation has thus far received the least amount of attention, likely because it is the newest antifungal drug class. Nevertheless, several studies have detected caspofungin-induced ROS in *C. albicans*, *N. glabratus*, and *A. fumigatus*, as well as micafungin-induced ROS in *C. albicans* and *N. glabratus* but not *A. fumigatus* (Belenky et al., 2013; Guirao-Abad et al., 2017; Satish et al., 2019; Garcia-Rubio et al., 2021) (**Table 1**). The mechanism(s) by which these drugs induce ROS formation are not known. Unlike azoles and polyenes, which target membranes, echinocandins target the cell wall and are thus not expected to disrupt mitochondria directly. Nevertheless, it has been proposed that fungi activate the PCD pathway in response to echinocandin-caused cell wall damage and that ROS are a key part of this PCD (Hao et al., 2013; Garcia-Rubio et al., 2021).

## Chemical nature of antifungal drug-induced ROS

As mentioned above, the term “ROS” encompasses a large number of molecules with oxidizing capacity, but their chemical nature, half-life, reactivity, diffusibility, and other properties vary widely. For instance, hydrogen peroxide H_2_O_2_ has low reactivity with cellular macromolecules but is capable of passing through cellular membranes and is therefore thought to function in signaling more than in oxidative damage (Winterbourn, 2008; Finkel, 2011; Sies et al., 2017; Sies et al., 2022). In contrast, the hydroxyl ion ^•^OH cannot diffuse across membranes but is highly and indiscriminately reactive, rapidly and irreversibly oxidizing proteins, lipids, and DNA in its vicinity, and is therefore highly toxic to cells (Sies et al., 2017; Sies et al., 2022). On the other hand, superoxide O_2_^•-^ is selectively reactive, preferentially oxidizing enzymes that contain iron-sulfur clusters (Sies et al., 2017; Sies et al., 2022). Thus, the functional consequences of the ROS induced in fungal cells in response to antifungal drugs depend on which specific ROS molecules are being produced.

The idea that different ROS molecules may be induced in different fungi and by different antifungals is supported by functional examinations undertaken in some of the studies cited above and in **Table 1**. For instance, Ferreira et al. studied ROS formation in *C. gattii* in response to amphotericin B and itraconazole and showed that although amphotericin B induced the greatest increase in overall ROS levels, itraconazole induced the greatest increase both in the levels of lipid oxidation and in the activities of superoxide dismutase and peroxidase, two enzymes involved in detoxifying ROS (Ferreira et al., 2013). Thus, the specific ROS induced by itraconazole may have had stronger oxidative or damaging capacity than the ROS induced by amphotericin B. Similarly, Shekhova et al. found that although amphotericin B caused the greatest accumulation of ROS in *A. fumigatus*, itraconazole resulted in higher lipid peroxidation (Shekhova et al., 2017). On the other hand, amphotericin B resulted in much greater mitochondrial membrane peroxidation and was also the only drug to elicit the nuclear translocation of Yap1, a conserved transcription factor activating oxidative response genes. Additionally, the same study reported that although the echinocandin caspofungin also elicited robust ROS induction (at least as high as amphotericin B), no mitochondrial membrane peroxidation was observed during caspofungin treatment (lipid peroxidation and Yap1 localization were not reported for caspofungin) (Shekhova et al., 2017). Furthermore, although in *C. parapsilosis* itraconazole induced ROS production, there was no concomitant increase in the activities of either catalase or superoxide dismutase, two of the primary enzymes responsible for detoxifying cellular ROS (Muñoz-Megías et al., 2023). Finally, we have reported that although the echinocandins caspofungin and micafungin elicit robust ROS induction in *N. glabratus*, we detected no concomitant increases in lipid peroxidation or induction of oxidative genes (Garcia-Rubio et al., 2021). Together, these and other results strongly suggest that the chemical nature and cellular consequences of the ROS induced by antifungal agents vary greatly, and that to understand their functions, it is first essential to discover their identity.

Unfortunately, the examination of ROS is challenging due to their unstable nature, short lifetimes, and high reactivity. As such, these molecules are rarely analyzed directly but are detected indirectly by measuring the products of their oxidant activity, usually by utilizing small molecules that become fluorescent upon oxidation. Indeed, virtually all studies reporting ROS formation in fungal cells have relied on fluorescence-based ROS detection methods using fluorescein-derived probes, such as dihydrofluorescein diacetate (DFH) and 2’,7’-dichlorodihydrofluorescein diacetate (H2DCFDA), whose levels can be measured by flow cytometry. Although these probes have the advantage of being easy to use, they lack specificity as they can be oxidized by a number of different ROS molecules; thus, an increase in fluorescence does not reveal which species are responsible for it (Zhang et al., 2018; Murphy et al., 2022). The same lack of specificity also characterizes other fluorescent probes used to measure ROS, such as dihydroethidium (DHE) and rhodamine-based dyes (Zhang et al., 2018; Murphy et al., 2022). A recently published consensus statement providing guidelines for measuring ROS in cells lists a number of alternative methods – all more labor-intensive and often requiring specialized equipment but also more unambiguous in their ROS identification (Murphy et al., 2022). One such method is electron spin resonance (ESR) (a.k.a., electron paramagnetic resonance), which uses small molecule spin traps that capture oxygen radicals, forming covalent bonds and stabilizing them, followed by detection by electron spin resonators (Murphy et al., 2022). Depending on the capabilities of the ESR instrument, different initiating ROS molecules can produce different ESR spectra, thus enabling the identification of the ROS present in cells. ESR has been successfully applied to detect and identify the ROS induced in bacterial cells by antibiotics (Thomas et al., 2013; Thomas et al., 2014; Thomas et al., 2015), suggesting that it can likewise be used to identify antifungal drug-induced ROS in fungal cells. Because ESR requires specialized and expensive equipment, it is usually not feasible for biologists to run these experiments in house. Some avenues for overcoming this challenge are collaborating with ESR experts in the chemistry and physics departments of nearby universities and research centers and taking advantage of shared ESR resource facilities (see, e.g., https://www.niehs.nih.gov/research/resources/epresr/resources/index.cfm).

For detecting non-radical intracellular ROS, such as H_2_O_2_, one can employ genetically encoded chimeric proteins, such as HyPer or roGFP-Orp1, in which a fluorescent protein is fused to, and regulated by, a H_2_O_2_-sensing protein from either bacteria (HyPer) or yeast (roGFP-Orp1) (Smolyarova et al., 2022). Indeed, a version of HyPer has been successfully used in the fungus *Fusarium* to detect intracellular fluctuations in H_2_O_2_ during cell division and differentiation (Mentges and Bormann, 2015), suggesting that it can also be used to measure H_2_O_2_ levels during antifungal drug treatment. These reporter constructs can be easily obtained (e.g., from https://www.addgene.org/) and adapted to one’s favorite fungal experimental system using standard molecular genetic techniques. In sum, the use of these and other methods capable of precisely identifying specific ROS molecules, such as liquid chromatography-mass spectrometry (LC-MS) and optical spectroscopy (Zhang et al., 2018; Murphy et al., 2022), will reveal the chemical nature of antifungal drug-induced ROS, which in turn will inform on their cellular functions.

## Cellular origins of antifungal drug-induced ROS

As mentioned above, ROS can be produced by cells in multiple ways. Innate immune cells contain dedicated enzymes, such as NADPH oxidases and NO synthases, which produce the ROS bursts aimed at attacking engulfed microbes. Fungal cells also contain NADPH oxidases, one of which, Fre8, has been implicated in producing ROS bursts during hyphal morphogenesis in *C. albicans* (Rossi et al., 2017). Whether fungal NADPH oxidases are involved in antifungal drug-induced ROS has not yet been investigated. ROS can also be derived from peroxisomes, which generate ROS during fatty acid oxidation and other lipid metabolic reactions (Antonenkov et al., 2010), and from the endoplasmic reticulum (ER) during protein folding, wherein one molecule of hydrogen peroxide is generated for each disulfide bond formed (Zeeshan et al., 2016; Rashdan and Pattillo, 2020). Thus far, the involvement of peroxisomes in antifungal drug-induced ROS formation has not been investigated. However, Yu et al. have provided evidence that in *C. albicans* the ER is involved in ROS formation in response to cell wall perturbation by Calcofluor White, a chitin binding compound (Yu et al., 2016). Thus, it is possible that echinocandins, which target cell wall integrity, also induce ROS via inducing ER stress; however, this remains to be experimentally verified. Finally, the major source of ROS in fungal cells is thought to be the mitochondria, where superoxide is formed as a result of electron leakage from respiratory complexes I and III during electron transfer (Mailloux, 2020; Yaakoub et al., 2022). The majority of this superoxide is rapidly converted into hydrogen peroxide by the action of mitochondrial superoxide dismutases (Sies et al., 2017; Warris and Ballou, 2019; Lennicke and Cochemé, 2021). Both superoxide and hydrogen peroxide can either remain inside the mitochondria or diffuse to the cytosol where they are neutralized by cytosolic superoxide dismutases and catalases, respectively (Sies et al., 2017; Warris and Ballou, 2019; Lennicke and Cochemé, 2021). Thus far, several studies have proposed that antifungal drug-induced ROS is predominantly of mitochondrial origin, but, as discussed below, this may not be true in every case.

Several studies have explicitly posited that the ROS induced by antifungal drugs are of mitochondrial origin (Mesa-Arango et al., 2014; Shekhova et al., 2017; Muñoz-Megías et al., 2023). A key piece of evidence supporting that conclusion was that the addition of rotenone, an inhibitor of mitochondrial complex I, significantly reduced the levels of ROS induced by amphotericin B and itraconazole in *A. fumigatus* (Shekhova et al., 2017), itraconazole in *C. parapsilosis* (Muñoz-Megías et al., 2023), and amphotericin B in *C. tropicalis* (Mesa-Arango et al., 2014). All of these fungi contain the canonical mitochondrial complex I, so the effect of rotenone could be explained due to its known inhibition of that complex, resulting in reduced electron leakage and ROS formation. Interestingly, however, rotenone also significantly reduced echinocandin-induced ROS formation in *N. glabratus* (Garcia-Rubio et al., 2021), which, like its close relative baker’s yeast *Saccharomyces cerevisiae*, lacks mitochondrial complex I, whose functions are carried out by rotenone-insensitive NADH-quinone oxidoreductases (Joseph-Horne et al., 2001; Schikora-Tamarit et al., 2021). Thus, in *N. glabratus* rotenone must be reducing echinocandin-induced ROS formation via another mechanism, and this mechanism cannot be ruled out in complex I-containing fungi as well. It is also possible that different antifungals induce ROS via different mechanisms, with membrane-targeting drugs (azoles and polyenes) acting primarily via the mitochondria but cell wall-targeting drugs (echinocandins) acting, for example, via the ER, as discussed above. Support for this hypothesis can be found in the study by Shekhova et al., who showed that in *A. fumigatus* ROS formation induced by amphotericin B and itraconazole resulted in mitochondrial membrane peroxidation and was reduced by rotenone, whereas neither of those effects were observed for the ROS induced by the echinocandin caspofungin (Shekhova et al., 2017). Future studies combining genetic mutations perturbing the functions of mitochondria, ER, peroxisomes, and NADPH oxidases with high resolution imaging (e.g., using ROS-sensitive genetically encoded probes mentioned above) will help conclusively identify the origins of the ROS induced by antifungal drug treatment.

## Cellular functions of antifungal drug-induced ROS

The ultimate questions regarding antifungal drug-induced ROS concern their cellular functions, i.e., the consequences of their formation, and whether and how this information can be leveraged to improve the efficacy of antifungal therapies. Broadly, the hypotheses regarding their functions can be classed into two categories: one where the ROS mediate or promote the antifungal mechanism of action, e.g., via causing cellular damage and facilitating cell death, and another, where the ROS are an adaptive response of the fungus to the drug because they have beneficial functions in that situation. These two hypotheses are not mutually exclusive, and both may be true depending on the specific drug-fungus context. Thus far, there is significantly more evidence for the first hypothesis, but our recent results (Garcia-Rubio et al., 2021; Arastehfar et al., 2023) and several recent reports on the role of ROS in stress-induced mutagenesis and the evolution of antibiotic-resistant mutants in bacteria (Pribis et al., 2019; Carvajal-Garcia et al., 2023) raise the possibility that antifungal drug-induced ROS may also mediate the evolution of antifungal drug resistance.

The association of ROS with fungal cell death has been noted in several studies showing that robust ROS production is induced by fungicidal but not fungistatic drugs (Francois et al., 2006; Belenky et al., 2013). However, in a few cases ROS have also been detected in response to fungistatic drugs (Mahl et al., 2015; Peng et al., 2018), and more work is necessary to understand the meaning of those results. The idea that ROS participate in the fungal killing by antifungal drugs has been suggested by a number of studies where pretreatment with ROS scavengers reduced antifungal efficacy and improved fungal survival. For instance, N-acetylcysteine antagonized the cidal activity of itraconazole in *C. albicans* (Lee and Lee, 2018) and amphotericin B in *A. fumigatus* (Shekhova et al., 2017), peroxynitrite scavenger FETPPS reduced *C. gattii* killing by amphotericin B (Ferreira et al., 2013), and thiourea reduced the killing of *C. albicans* by amphotericin B and micafungin (Guirao-Abad et al., 2017). In all of these experiments, the antifungal agents were used at the MIC or above-MIC concentrations, inducing a high degree of cell killing. These results are consistent with two alternative explanations. First, the ROS may be induced directly by the activity of the antifungal drug, e.g., via disruption of mitochondrial membrane or inducing ER stress, and these ROS then damage cellular components, promoting cell death. Second, the antifungal drug may trigger PCD in the fungus, and the ROS would then form as part of the apoptotic cascade, irrespective of the original antifungal mechanism of action. In support of the second hypothesis, several studies have provided evidence that fungal cells exposed to fungicidal drugs exhibit multiple features of PCD besides ROS formation, such as plasma membrane phosphatidylserine externalization, caspase activation, and DNA fragmentation (Phillips et al., 2003; Hao et al., 2013; Lee and Lee, 2018). In either scenario the ROS would damage the cell, but in the first case, the ROS would be part of the primary drug-induced damage, whereas in the second case, ROS-induced damage would be secondary to the drug-induced damage. How these two possibilities could be distinguished has been demonstrated by elegant experiments conducted in bacteria, where it was shown that addition of a ROS scavenger after the removal of a bactericidal antibiotic (i.e., when the cells are no longer experiencing primary antibiotic-induced damage) significantly improves bacterial survival, indicating that the ROS are part of a secondary death-triggering cascade (Hong et al., 2019). Similar experiments may help decipher the role of ROS in antifungal drug-induced fungal cell death.

Our recent studies have hinted at another possible role for ROS formed in response to echinocandin action. We detected robust ROS induction in *N. glabratus* during treatment with caspofungin and micafungin (Garcia-Rubio et al., 2021). We tested several compounds with ROS scavenger activity, and only pre-incubation with ascorbic acid significantly reduced this increase in ROS. Interestingly, however, ascorbic acid had no effect on *N. glabratus* killing by caspofungin, suggesting that the ROS were not participating in cell death. Consistent with that conclusion, when we deleted several enzymes involved in ROS detoxification, including catalase and superoxide dismutase, the ROS levels increased, but lethality was not altered (Arastehfar et al., 2023). However, the ROS detoxification mutants showed a several orders of magnitude increase in the emergence of echinocandin-resistant mutations, suggesting that the ROS may promote mutagenesis in drug-treated cells. This conclusion is consistent with an emerging picture in bacteria, where several recent discoveries have pointed to a key role for ROS in stress-induced mutagenesis and the evolution of antibiotic-resistant mutants (Pribis et al., 2019; Pribis et al., 2022; Carvajal-Garcia et al., 2023). In particular, treatment of *E. coli* with sublethal quinolone doses induces ROS in a subset of treated cells (Pribis et al., 2019). These ROS do not damage DNA but induce the general stress response, which in turns upregulates mutagenesis, leading to mutations that cause resistance to quinolones as well as other antibiotics not yet encountered by the bacteria (Pribis et al., 2019). Furthermore, another recent study showed that in several bacterial species *in vitro* evolution of resistance to multiple antibiotics is significantly slowed down in the presence of ROS scavenger thiourea (Carvajal-Garcia et al., 2023). It was also shown that the ROS-linked mutagenesis was mediated by enzymes involved in transcription-coupled repair, nucleotide excision repair, as well as several error-prone DNA polymerases. Together, these studies identify the various ways in which ROS can promote the evolution of drug-resistant mutations, and similar mechanisms may operate in fungal cells. Finally, as another example of a beneficial role of antifungal drug-induced ROS, we have shown that caspofungin-induced ROS in *A. fumigatus* promote the acquisition of echinocandin tolerance via non-genetic mechanisms involving altered plasma membrane lipid microenvironment of the echinocandin target, 1,3-β-glucan synthase enzyme (Satish et al., 2019).

## ROS and stress-induced cell death in fungi

The strong association between antifungal drug cidality and ROS formation fits well within the broader paradigm of stress-induced fungal cell death (Grosfeld et al., 2021), two major types of which have been described: PCD (a.k.a., apoptosis) and necrosis (Ramsdale, 2008; Strich, 2015). Both necrosis and PCD are associated with ROS formation (Eisenberg et al., 2010; Strich, 2015), and both have been detected in fungal cells treated with antifungal drugs (Phillips et al., 2003; Mousavi and Robson, 2004; Hao et al., 2013). For example, *A. fumigatus* treated with lower doses of amphotericin B exhibited multiple features of PCD, whereas treatment with higher doses led to necrosis (Mousavi and Robson, 2004). PCD has many similarities with mammalian apoptosis and is defined as a programmed, active cellular response to damage, characterized by mitochondrial membrane permeabilization, ROS accumulation, chromatin condensation, nuclear DNA fragmentation, externalization of plasma membrane phosphatidylserine, and activation of pro-death proteins such as homologs of mammalian caspases. In contrast, necrosis is defined as damage-induced loss of cellular integrity, in which the cell is less of an active participant. It is thought that in unicellular eukaryotes PCD functions to eliminate damaged cells from the population to make more resources available for undamaged ones, which is consistent with antifungal drug-induced damage activating PCD. ROS are thought to promote macromolecular damage and to facilitate cellular demise; thus, inclusion of ROS scavengers can alleviate stress-induced lethality (Ramsdale, 2008), which, as discussed above, is also the case for several antifungal drugs. With respect to the cellular origins of PCD-associated ROS, although damaged mitochondria are often invoked as their source, ROS derived from the ER, peroxisomes, and NADPH oxidase Yno1 have also been implicated in fungal PCD (Strich, 2015).

ROS can act as both the trigger for PCD activation, e.g., in response to an external source of oxidative damage, such as H_2_O_2_, and as one of the features of PCD activated by a different stress, such as heat shock, acid stress, membrane stress, or cell wall stress (Grosfeld et al., 2021). This dual role makes it difficult to disentangle whether the ROS induced by antifungal drugs are a direct result of antifungal drug action (e.g., via mitochondrial membrane disruption), which in turn triggers PCD, or whether the ROS are a secondary consequence of PCD caused by a different type of antifungal-induced damage (e.g., to the cell wall). Indeed, the answer may be different for different drugs and different fungi, and these scenarios are not mutually exclusive. An intriguing question that has not, to our knowledge, been explored is whether the PCD program can be activated by fungal cells but not carried out to completion, instead utilizing PCD features (ROS formation, DNA fragmentation) to promote genome instability and evolvability in cells that survive drug exposure.

## Conclusion

The accumulating evidence for the role of ROS in medically-relevant fungi adds to the large body of literature demonstrating the importance of ROS in eukaryotic stress responses and in human health, including inflammation (Forrester et al., 2018), cancer (Poillet-Perez et al., 2015; Cheung and Vousden, 2022), metabolic diseases (Newsholme et al., 2016), and aging (de Almeida et al., 2022). As in those contexts, experimental evidence indicates that antifungal drug-induced ROS have important functions, whether in promoting cell death at lethal concentrations of antifungals, promoting mutagenesis and evolution of drug resistance at sublethal concentrations of antifungals, or other, as yet unknown, mechanisms. However, many key questions still remain regarding the chemical nature, molecular origins, and functional consequences of these ROS, as well as their connection to PCD. Decades of research and many dozens of studies focusing on analogous questions in bacteria have revealed profound mechanistic insights, which are being leveraged to identify new ways to potentiate antibiotic-mediated killing (Brynildsen et al., 2013; Jiang Q et al., 2020) or prevent the evolution of antibiotic resistance (Zhai et al., 2023). Thus, there is every reason to believe that gaining an understanding of the role of ROS in fungi treated with antifungal drugs will help develop new tools to fight deadly invasive fungal infections, as well as reveal a fascinating aspect of fungal biology.

## Author contributions

ES: Writing – original draft, Writing – review & editing. IG: Writing – original draft. DP: Writing – review & editing.
